# Dietary Interventions in Metabolic Dysfunction-Associated Steatotic Liver Disease: A Narrative Review of Evidence, Mechanisms, and Translational Challenges

**DOI:** 10.3390/nu17213491

**Published:** 2025-11-06

**Authors:** Alejandra Paredes-Marin, Yulu He, Xiaotao Zhang

**Affiliations:** 1 Division of Liver Diseases, Department of Medicine, Icahn School of Medicine at Mount Sinai, New York, NY 10029, USA; alejandra.paredesmarin@mssm.edu (A.P.-M.); lulu.he@mountsinai.org (Y.H.); 2 University of Pittsburgh Medical Center, University of Pittsburgh School of Medicine, Pittsburgh, PA 15260, USA; 3 Institute for Translational Epidemiology, Icahn School of Medicine at Mount Sinai, New York, NY 10029, USA; 4 Cancer Prevention and Control, The Tisch Cancer Institute, Icahn School of Medicine at Mount Sinai, New York, NY 10029, USA

**Keywords:** diet intervention, MASLD/MASH, steatosis, fibrosis, microbiome, nutrition

## Abstract

**Background/Objectives**: Metabolic dysfunction-associated steatotic liver disease (MASLD) is rapidly attracting growing concern around the world. While there has been progress in the development of pharmacologic treatments, lifestyle and dietary interventions remain as the first-line approach for management. This scoping review aimed to identify dietary strategies for managing MASLD and to highlight current research gaps and challenges. **Methods**: A systematic search of PubMed and Science Direct was conducted up to 10 July 2025, for relevant studies on dietary modifications and MASLD. Data extracted included types of interventions, outcomes related to liver health, and research limitations. **Results**: Dietary interventions were shown to consistently improve hepatic and metabolic outcomes. In a randomized controlled trial of 12 weeks (*n* = 259), a Mediterranean diet reduced hepatic steatosis by 39% and improved insulin sensitivity. A calorie-restricted lifestyle program in adults with MASLD (*n* = 196) reduced liver fat by 25% over 52 weeks. Resistant starch supplementation (*n* = 200) lowered intrahepatic triglyceride content by 8% through gut microbiome modulation. A pilot RCT of medically tailored meals in cirrhosis (*n* = 40) reduced ascites symptoms and improved quality of life. Finally, prebiotic supplementation in MASLD (*n* = 200) lowered systemic inflammation and increased immune-regulating microbes. In contrast, Western dietary patterns and ultra-processed foods were consistently linked to lipotoxicity and inflammation. **Conclusions**: Dietary interventions remain critical for the management of chronic liver disease and continue to play a vital role even as pharmacotherapy options emerge. Further research should explore precision nutrition and microbiome-based therapies while also addressing the methodological limitations like the underutilization of causal inference frameworks. Finally, it is also important to consider culturally tailored interventions to account for barriers in access and equity in underserved populations.

## 1. Introduction

Metabolic dysfunction-associated steatotic liver disease (MASLD), formerly known as nonalcoholic fatty liver disease (NAFLD), represents a growing global health issue, with an estimated prevalence in the world of approximately 24% [1]. MASLD and its more advanced form, metabolic dysfunction-associated steatohepatitis (MASH), have become the leading causes of chronic liver disease, alongside chronic hepatitis B, hepatitis C, and hepatocellular carcinoma (HCC). Alarmingly, projections indicate that the number of MASH cases in the United States will rise from 14.9 million adults (5.8%) in 2020 to 23.2 million (7.9%) by 2050 [2]. This increase is expected to result in a significant rise in liver-related complications, including HCC, liver transplantation, liver-related hospitalizations, acute liver failure, and overall progression of chronic liver disease [3].

While there has been incredible progress in the development of pharmacologic treatments for MASLD/MASH, important challenges remain. In 2024, the U.S. Food and Drug Administration (FDA) approved resmetirom (Rezdiffra) as the first therapy for MASH with moderate to advanced fibrosis (F2–F3) [4]. In 2025, the FDA also approved the GLP-1 receptor agonist semaglutide (Wegovy) for adults with noncirrhotic MASH and moderate–to–advanced fibrosis [5]. Dual GIP/GLP-1 receptor agonists (e.g., tirzepatide) remain under active investigation. However, these medications face real-world limitations including side effects, high costs, restricted access, and adherence issues [6]. Therefore, only a limited number of drugs have been specifically approved for MASLD or MASH, highlighting a continuous gap in therapeutic options [7].

Given these constraints, dietary interventions remain as first-line approaches for MASLD management [8]. These approaches are particularly critical in the early stages of disease and continue to play a vital role as adjuncts to pharmacotherapy, helping to enhance treatment efficacy, reduce adverse effects, and support long-term changes [6].

The purpose of this review is to evaluate the current evidence supporting dietary strategies for MASLD, assess their integration with emerging pharmacologic therapies such as resmetirom and GLP-1 receptor agonists, and identify priorities for future research and clinical application.

## 2. Methods

Since no previously registered review protocol existed, a new one was developed for this study. Eligibility criteria included peer-reviewed articles published in English or Spanish between 2010 and 2025 that were fully available online at the time of review.

Intervention studies were prioritized, and when unavailable, observational studies were included. Information sources consisted of PubMed and ScienceDirect, with searches conducted from 10 June to 22 September 2025. For example, a PubMed search for “NAFLD diet treatment” retrieved relevant studies on diet and MASLD, such as ‘Romero-Gómez et al. (2017)’ in Journal of Hepatology.

No limits were applied other than excluding studies published before 2010. As mentioned, the selection process prioritized interventional studies, followed by observational and cohort studies. Data charting did not include contacting investigators for confirmation. Synthesis of results involved summarizing the reported outcomes of the included studies and cohorts.

## 3. Dietary Interventions

Lifestyle medicine, particularly when centered on structured dietary interventions, represents the cornerstone of both the prevention and treatment of metabolic dysfunction-associated steatotic liver disease (MASLD). Contemporary evidence and international guidelines (EASL–EASD–EASO, 2024; AASLD, 2023) emphasize that comprehensive lifestyle modification—comprising dietary optimization, regular physical activity, and behavioral support—is the first-line strategy capable of halting or even reversing hepatic steatosis and early fibrosis [9,10]. Sustained weight reduction of 7–10% is consistently associated with histologic improvement and normalization of aminotransferases [11]. Among dietary models, the Mediterranean-style diet has the most robust evidence, improving hepatic fat content, insulin sensitivity, lipid profiles, and systemic inflammation, while simultaneously reducing cardiovascular risk [12,13,14]. Adjunctive dietary strategies—such as moderate carbohydrate restriction, higher protein intake, and time-restricted eating—may further enhance metabolic and hepatic outcomes when individualized to patient preference and cardiometabolic context [15,16]. For carefully selected non-diabetic adults with biopsy-proven MASH, vitamin E can reduce steatosis and lobular inflammation, though antifibrotic effects remain inconsistent [10,17]. By targeting central drivers of disease, including visceral adiposity, insulin resistance, dyslipidemia, and systemic inflammation, lifestyle medicine offers an integrated framework to prevent MASLD progression and mitigate associated cardiovascular complications [9,10].

### 3.1. Dietary Patterns and Nutritional Interventions

Dietary interventions remain as first-line approaches for managing MASLD-, MASH-, and MASH-related fibrosis. Evidence from real-world studies consistently emphasize the efficacy of targeted nutritional strategies in reducing hepatic steatosis, improving metabolic parameters, and slowing disease progression. As pharmacologic options continue to be developed, dietary patterns and nutrient-specific approaches continue to offer a practical, accessible, and sustainable cornerstone of care—particularly in early disease stages where most of the current drugs are still not recommended.

#### 3.1.1. Mediterranean Diet and Plant-Based Approaches

Among the various dietary strategies, the Mediterranean diet (MD) has become a reference model in MASLD and MASH management. This diet is characterized by a high intake of whole grains, fruits, vegetables, legumes, nuts, olive oil, and moderate consumption of fish and poultry. Also, the MD is known to be rich in monounsaturated (MUFA) and polyunsaturated fatty acids (PUFA) and low in red meat, refined grains, saturated fats, and trans fats. This diet not only promotes weight loss, but dietary interventions have also been shown to reduce intrahepatic fat accumulation, oxidative stress, and insulin resistance, key contributors to MASLD pathophysiology [18,19,20]. Notably, an 18-month RCT (DIRECT-PLUS) testing a polyphenol-enriched ‘green-MD’ (MD + walnuts, green tea, Mankai) showed greater intrahepatic fat reduction than standard MD and healthy-diet guidance (≈−39% vs. −20% vs. −12%), supporting composition-specific benefits of a plant-forward MD variant [21].

A study that used cluster analysis showed that plant-based approaches have similar anti-inflammatory effects, and fiber-rich foods have shown promise in improving metabolic outcomes associated with liver and gut health [22].

#### 3.1.2. Western Diets and Hepatotoxicity

In contrast, Western diets are characterized by high intakes of meat-derived fats and refined carbohydrates and low consumption of fruits and vegetables. These diets have been shown in multiple randomized controlled trials to be associated with worse liver outcomes [2,23,24]. Western diets contribute to metabolic dysregulation, lipotoxicity (lipid accumulation in non-adipose tissues that leads to cellular dysfunction), and hepatic inflammation, increasing the risk of progression from MASLD to MASH and ultimately to cirrhosis or HCC.

#### 3.1.3. Ketogenic/Low-Carbohydrate Diet

Ketogenic diets (KD)—very-low-carbohydrate, high-fat regimens that induce nutritional ketosis—have recently attracted attention as potential metabolic interventions for MASLD. Short-term clinical trials and meta-analyses demonstrate that KDs can substantially reduce intrahepatic lipid content, improve insulin sensitivity, and lower serum triglycerides, largely through rapid mobilization of hepatic and visceral fat stores and enhanced mitochondrial β-oxidation [25,26,27,28,29]. In some studies, hepatic fat reductions of up to 30–50% (by MRI-PDFF) have been observed within 8–12 weeks, even independent of significant weight loss [26]. However, evidence also indicates heterogeneity in response, and longer-term adherence and cardiometabolic safety remain concerns. Elevated LDL-cholesterol and potential nutrient deficiencies have been reported, underscoring the need for individualized dietary supervision and lipid monitoring [30,31,32]. While ketogenic diets may serve as short-term, targeted metabolic therapy for selected adults with obesity, insulin resistance, or uncontrolled hyperglycemia, they should ideally transition toward more sustainable, cardioprotective patterns—such as the Mediterranean or moderate-carbohydrate diets—once hepatic and metabolic control is achieved [33]. Further randomized trials are warranted to clarify the durability, safety, and histologic impact of KDs in diverse MASLD phenotypes.

#### 3.1.4. Ultra-Processed Foods and MASLD

Ultra-processed foods (UPFs) are industrially formulated products made mostly or entirely from substances extracted from foods (e.g., oils, fats, sugars, starches, and proteins), derived from food constituents (e.g., hydrogenated fats, modified starch), or synthesized in laboratories (e.g., flavor enhancers, colorants, emulsifiers). They typically contain little to no whole food and are designed to be hyper-palatable, convenient, and shelf-stable [34]. In the context of liver disease, studies have shown that high consumption of UPFs is associated with increased risk of developing and progressing metabolic dysfunction–associated steatotic liver disease (MASLD) due to their high energy density, poor nutritional quality, and potential to disrupt gut–liver axis homeostasis [35]. A longitudinal cohort study assessed dietary intake and lifestyle factors using a validated food frequency questionnaire, with food items classified according to the NOVA system. Participants were grouped based on changes in UPFs consumption. The study found that reducing UPFs intake significantly lowered intrahepatic fat content and reduced calorie intake [36].

In another study that surveyed patients with MASLD, they were more likely to report dietary habits characterized by high intake of processed foods rich in added sugars, salt, unhealthy fats, and high-fructose corn syrup, as well as frequent consumption of fast food and meals outside the home [2,37]. These dietary patterns were linked to increased insulin resistance, gut dysbiosis, and systemic inflammation, all key mechanisms in MASLD pathogenesis [37].

#### 3.1.5. Nutrient-Specific Interventions

Several nutrients have been evaluated for potential benefit or harm in liver disease. Vitamin E has antioxidant effects that may attenuate hepatic inflammation and steatosis in non-diabetic adults with biopsy-confirmed MASH [38]. The PIVENS randomized, double-blind, placebo-controlled trial demonstrated that vitamin E at 800 IU/day for 96 weeks improved key histologic features of MASH such as steatosis, lobular inflammation, and ballooning, in non-diabetic, biopsy-proven patients, though an antifibrotic effect was not evident. Consequently, current guidance supports vitamin E for carefully selected non-diabetic adults without cirrhosis, using shared decision-making about dose and duration given mixed long-term safety signals in other populations [39].

Omega-3 fatty acids, particularly marine n-3 PUFAs, have also shown promise. In a six-month RCT in MASLD, fish-oil supplementation improved lipid profiles and was associated with reductions in liver fibrosis as measured by vibration-controlled transient elastography (FibroScan 502 Touch; Echosens, Paris, France). Measurements were obtained with M or XL probes per manufacturer guidance [40]. Across meta-analyses and RCTs in NAFLD/MASLD, omega-3 PUFAs consistently reduce liver fat on imaging and lower triglycerides, with favorable effects on overall lipid profiles [41]. However, effects on fibrosis, liver stiffness, and histology are inconsistent or modest across many trials [42].

Collectively, these data suggest that in MASLD/MASH, the quality of dietary fat, emphasizing unsaturated fats (MUFA + PUFA) over saturated fats and an appropriate n-6:n-3 PUFA balance, warrants particular attention, while additional high-quality trials are needed to clarify effects on fibrosis and long-term outcomes.

#### 3.1.6. Food-Specific Interventions

Recent research has also begun to highlight the potential of food-specific interventions in MASLD. Foods such as beans and resistant starches have shown promise due to their beneficial effects on the gut microbiome, systemic inflammation, and glucose metabolism. A four-month, randomized, double-blind trial had 196 adults undergo supplementing 40 g/day of resistant starch or an isocaloric control starch. The first group observed a 9.0% absolute reduction in intrahepatic triglyceride content. These results were linked to alterations in gut microbiota and lower serum branched-chain amino acids [43].

Moreover, a randomized trial of 55 patients found that bean consumption has prebiotic effects and the ability to enhance the gut microbiome and regulate host biomarkers linked to metabolic obesity and colorectal cancer [44]. These properties make them attractive adjuncts in the dietary management of MASLD, particularly in targeting underlying mechanisms such as insulin resistance and low-grade inflammation [45].

## 4. Meal Timing—Time-Restricted Eating (TRE)

Two randomized trials inform TRE in MASLD/NAFLD. First, a 12-week single-blind AB/BA crossover trial in adults with NAFLD tested 16:8 TRE without prescribed caloric restriction versus standard lifestyle advice. TRE significantly reduced hepatic steatosis by CAP (mean −9.96 vs. +20.46 dB/m; between-intervention *p* = 0.038) and also lowered body weight and waist circumference despite no mandated energy deficit, with larger CAP improvements among participants with baseline CAP ≥ 268 dB/m [46].

Second, the 12-month TREATY-FLD randomized clinical trial compared early-time TRE (8:00–16:00) with matched daily caloric restriction to daily caloric restriction alone. Both groups achieved similar reductions in intrahepatic triglyceride (IHTG) content at 6 and 12 months (TRE −6.9% vs. DCR −7.9% at 12 months; between-group NS) and comparable improvements in liver stiffness, weight, and metabolic risk factors—implicating energy restriction as the principal driver of benefit when calories are controlled [47].

Implication for MASLD: TRE appears feasible and can improve hepatic steatosis and adiposity when used ad libitum (no mandated calorie cut), potentially via circadian alignment and spontaneous intake reduction; however, when caloric intake is equalized, TRE offers no additional liver-specific advantage over conventional calorie restriction. Clinically, TRE may be positioned as an adherence-friendly option to induce or support energy reduction while also aligning eating with circadian biology.

## 5. Body Weight Reduction

Body weight reduction remains a pivotal therapeutic target in MASLD, as even modest losses confer substantial improvements in hepatic steatosis and metabolic health. In a 52-week randomized trial conducted in Singapore among obese, non-diabetic adults with MRI-confirmed NAFLD, participants were assigned to either a supervised diet–exercise program (≈400 kcal/day energy deficit plus 150–200 min/week moderate exercise) or liraglutide 3 mg/day for 26 weeks, followed by a 26-week observation period. Both strategies produced comparable reductions in body weight (−3.5 ± 3.3 kg vs. −3.0 ± 2.2 kg), liver fat fraction (−8.1 ± 13.2% vs. −7.0 ± 7.1%), and ALT/AST levels at 26 weeks. However, only liraglutide significantly decreased high-sensitivity CRP, indicating an additional anti-inflammatory effect beyond weight loss. Upon discontinuation, participants previously receiving liraglutide experienced partial rebound in weight, liver fat, and hepatocyte apoptosis marker (cCK-18), whereas those in the lifestyle arm maintained improvements—highlighting the importance of continued behavioral modification for sustained metabolic benefit [48].

Building on these findings, a more recent randomized experimental-medicine trial [49] investigated liraglutide vs. lifestyle modification in adults with MASLD without type 2 diabetes. Over 12 weeks of treatment followed by 12 weeks of post-treatment observation, both groups achieved matched, clinically meaningful weight loss, resulting in comparable reductions in liver fat (MRS), iron-corrected T1, and ALT. The liraglutide group exhibited greater improvements in glucose handling, fasting lipids, and suppression of de novo lipogenesis, though post-withdrawal proteomic changes suggested a potential for weight regain.

Implication: Across both trials, comparable hepatic benefits were observed when weight loss was equivalent, regardless of whether achieved pharmacologically or through lifestyle modification—underscoring weight reduction itself as the primary determinant of liver improvement in MASLD.

## 6. Microbiome

Emerging research on the role of the microbiome suggests that it may serve as a key mechanistic link between lifestyle factors and liver health. This has offered a promising new field for future interventions. The microbiome is now recognized as an important contributor to the development and progression of MASH, influencing both liver injury and recovery.

### 6.1. Prebiotics and Probiotics

Prebiotic fibers are nondigestible carbohydrates that modulate the gut microbiota toward a composition beneficial to the host. A very recently published pilot randomized controlled trial enrolled patients aged 18–70 with MASLD and metabolic syndrome, diagnosed by ultrasound and with ALT ≥ 30 U/L—received prebiotic supplementation while maintaining stable weight [50]. Although treatment significantly increased the relative abundance of fecal *Bifidobacterium*, it did not produce favorable effects on liver fat content, hepatic or metabolic parameters, inflammatory markers, or FGF-19 levels. These findings align with prior studies suggesting that prebiotic supplementation without concurrent weight loss is insufficient to improve liver or metabolic outcomes in MASLD.

Probiotic organisms have also been shown to exert beneficial effects on the gut microbiota and host metabolism. In a randomized, double-blind, placebo-controlled trial (*n* = 68), participants received a mixture of six probiotic strains (*Lactobacillus acidophilus*, *L. rhamnosus*, *L. paracasei*, *Pediococcus pentosaceus*, *Bifidobacterium lactis*, and *B. breve*) for 12 weeks. These strains are among the most commonly used in studies of obesity and fatty liver disease. The main outcome demonstrated that mean intrahepatic fat, measured by MRI-PDFF, decreased from 16.3% to 14.1% in the probiotic group (mean difference vs. placebo ≈ −2.6%, *p* ≈ 0.013) [51].

### 6.2. Synbiotics

Combining the effects of both prebiotics and probiotics has also been explored in recent studies evaluating their impact on MASLD. A meta-analysis of 10 randomized controlled trials involving 634 patients with MASLD systematically investigated the effects of synbiotic supplementation. The results demonstrated that synbiotic significantly reduced several liver outcome markers (ALT, AST), including liver stiffness [52]. Overall, the meta-analysis suggests that synbiotic supplementation can improve liver function, modulate lipid metabolism, and reduce liver fibrosis in patients with MALSD; however, these findings require confirmation in larger, well-controlled studies

### 6.3. Microbiota Consortia and Fecal Microbiota Transplantation (FMT)

Like probiotics, which typically contain one well-characterized strain, microbiota consortia are designed communities of microorganisms intended to mimic or restore the function of a healthy gut microbiota. These consortia are synthetic, defined mixtures of microorganisms, whereas fecal microbiota transplantation (FMT) involves transferring the entire microbiota from a healthy donor. While FMT has been studied in MASLD, there is currently limited research on microbial consortia in humans, it is necessary to investigate their potential effects.

In a randomized controlled trial, patients with MASLD were assigned to receive either FMT (via colonoscopy followed by three enemas over three days) or oral probiotics. Both groups were advised to maintain a healthy diet and engage in at least 40 min of daily exercise. One month after treatment, patients were re-evaluated. The authors reported that FMT reduced hepatic fat accumulation compared with the probiotic control group [53].

### 6.4. The Gut–Liver Axis and Oral–Gut–Liver Axis: Role of the Microbiome

Beyond the classic gut–liver axis, one complementary pathway is increasingly recognized:

- Oral–gut–liver axis: dysbiotic oral communities seed or perturb the intestinal microbiome, which in turn affects barrier integrity, microbial metabolites, and immune tone that converge on the liver [54,55,56].

In MASLD/MASH, oral dysbiosis can seed and perturb the gut microbiota, impair epithelial barrier function, and increase intestinal permeability; resultant microbial products (e.g., endotoxin/LPS) enter the portal circulation, amplifying hepatic inflammation and fibrogenesis [56]. As an applied example of this oral–gut–liver pathway, a multicenter RCT in MASLD patients with periodontitis showed that intensive periodontal therapy (scaling and root planing) reduced ALT and *P. gingivalis* antibody titers over 12 weeks, consistent with attenuated hepatic inflammatory signaling [57].

Implications for research and care. These axes suggest testable, adjunctive strategies: (i) integrating periodontal assessment and treatment into MASLD care pathways; (ii) targeting oral keystone pathogens and salivary dysbiosis alongside gut-focused therapies; (iii) incorporating barrier integrity readouts and microbial/metabolite biomarkers to track liver–microbiome crosstalk; and (iv) designing longer, well-controlled trials to determine whether sustained oral health interventions translate into histologic or stiffness improvements beyond short-term enzyme changes.

### 6.5. Multi-Omics and Precision Nutrition

Research on the human microbiome is now also moving from isolated, single-parameter analyses to more comprehensive multi-omics approaches. By integrating genomics, metabolomics, and epigenomics, scientists are looking to better understand the complex functional roles of the gut microbiota in MASLD/MASH. Genomic data allows for the study of host–microbiome interactions, metabolomic profiling provides insights into the bioactive compounds produced by gut microbes, and epigenomic analyses reveal how gene regulation may be altered in disease states [58].

A study used this multidimensional approach and combined metagenomic and metabolomic studies to identify short-chain fatty acids (SCFAs), produced by gut microbes, as key regulators of hepatic lipid metabolism and immune responses. These microbial metabolites not only influence MASLD progression but also present opportunities for novel therapeutic targets. Moreover, other microbial-derived compounds such as glycine-conjugated secondary bile acids (GCS) have emerged as potential biomarkers for MASH, offering paths for microbiota-based diagnostics and treatment strategies [59].

Although these are promising studies, there still remains a lack of large-scale human clinical trials and longitudinal studies applying these techniques. There is a need for more evidence and data on the human microbiome using multi-omics approaches and metabolomic analysis. Filling this gap could significantly advance the development of precision nutrition strategies and microbiome-based interventions tailored to individual patients’ biological profiles.

A summary of dietary, weight reduction and microbiome interventions on MASLD/MASH is shown in Table 1.

## 7. Methodological Limitations and Causal Inference

As mentioned previously, there is a gap of scientific evidence for dietary interventions in chronic liver diseases. This lack of evidence-based approaches is usually limited by the following:

- Observational study bias: Most findings come from non-randomized studies, which are prone to confounding, placebo effects and reverse causality [60].
- Short intervention duration: Many interventions only last a short amount of time, limiting insight into long-term effects or histologic progression. A systematic review found that most clinical trials evaluating diet and exercise in MASLD last between 8 and 24 weeks, and only a few interventions extended beyond 24 weeks [61]. Additionally, a randomized trial of postmenopausal women with biopsy-confirmed MASLD showed that a 24-week exercise intervention results in metabolic benefits but not significant changes in liver fat. This is especially important because it shows that these shorter trials may fail to capture the full extent of hepatic improvements [62].
- Underutilization of causal frameworks: Tools such as target trial emulation are not widely applied but have great potential to improve causal inference from observational data.

Forward-looking solutions. To strengthen the evidence base while remaining feasible and patient-centered, we propose the following:

**Mitigating observational bias (within existing and new cohorts):**

- Pre-specify exposures, outcomes, and causal contrasts using directed acyclic graphs; apply active comparators, new-user designs, and rigorous confounding control (e.g., propensity methods, g-methods) [60,63].
- Incorporate negative-control exposures/outcomes and quantitative bias analyses (e.g., E-values) to probe residual confounding.
- Leverage Mendelian randomization and instrumental variable strategies, where valid instruments exist, to reduce confounding and reverse causality; prior bidirectional MR linking diet with liver outcomes illustrates this potential and underscores food groups (e.g., beans) as testable prebiotic interventions [64].

**Overcoming short duration and enhancing outcome capture:**

- Design **longer pragmatic trials** (≥48–96 weeks) with remote follow-up, wearables, and EHR linkage to maintain adherence and reduce cost.
- Use hybrid outcome strategies: short-term mechanistic endpoints (e.g., MRI-PDFF, MRE, serum biomarkers) embedded within longer follow-up to assess histology, fibrosis progression, and clinically meaningful events.
- Consider adaptive and platform features (response-adaptive randomization, interim futility) to focus resources on promising nutrient patterns (e.g., Mediterranean-style, higher MUFA/PUFA; n-6:n-3 balance) while maintaining rigor.
- Employ registry-based or cohort-embedded RCTs to accelerate recruitment and extend follow-up via routine care data.

**Systematizing causal frameworks:**

Normalize the use of target trial emulation (TTE) for key questions that are impractical to randomize at scale (e.g., sustained dietary patterns), alongside MR and instrumental variable analyses to triangulate evidence [63,64].Target trial emulation can strengthen inference in observational cohort by specifying, a priori, the key components of a hypothetical randomized trial—eligibility criteria (e.g., adults with MASLD without cirrhosis), treatment strategies (e.g., sustained adherence to a Mediterranean-style or PUFA-enriched dietary pattern vs. lower adherence), time zero (common start of follow-up), outcomes (e.g., liver fat change, fibrosis progression, metabolic endpoints), and follow-up and censoring rules. By aligning exposure classification to a grace period, using active comparators and new-user designs, and adjusting for baseline and time-varying confounders with g-methods, TTE reduces confounding and immortal-time bias, yielding more robust causal estimates from routinely collected cohort data [63].

## 8. Implementation and Equity Considerations

While dietary interventions are low-cost, their real-world implementation remains challenging due to several factors, including the sustainability of behavior change, access to healthy foods and resources, and the need for culturally tailored interventions.

Behavior change sustainability is a major challenge, as patients usually struggle to maintain long-term dietary modifications, essential for effective MASLD management. Adherence to both dietary changes and regular exercise is often difficult due to competing priorities, such as work and family obligations, which tend to take precedence over physical activity and healthy eating. In a representative sample of 2000 Polish adults, the authors reported the most frequently cited barriers to following a healthy diet as: high cost of healthy foods (43%), lack of motivation (26.7%), and insufficient time (25.4%) [65].

A pilot randomized control trial tested low-sodium, high-protein medically tailored meals in 40 patients with ascites and cirrhosis. They were successful in showing these medically tailored meals reduced the need for paracentesis and improved quality of life [66]. This intervention has the potential to become a low-cost, accessible therapeutic option that can be administered at home and reduce hospital visits. Even though evidence of medically tailored meals in MASLD/MASH is still emerging, it appears to be a very promising field with possible meaningful clinical benefits.

Access to healthy foods also poses significant challenges. One study found that individuals face multiple barriers, including the high cost of healthy options, difficulty adjusting eating habits such as reducing portion sizes, limited variety, and quality of food in local stores, and the challenge of avoiding unhealthy options at community events and social gatherings [67].

Cultural tailoring of interventions is therefore crucial for improving health outcomes and adherence, especially in diverse populations. Tailored lifestyle modification programs that respect cultural dietary preferences and leverage community-based support can improve engagement and effectiveness. Diet plans should be individualized, culturally sensitive, socially appropriate, and include foods that are readily available. Without this personalization, patients may struggle to follow recommended dietary guidelines, reducing the overall impact of the dietary intervention [68,69].

A scoping review that sought to assess the effect of nutritional interventions that incorporated culturally relevant topics into their design and analyses reviewed 20 community interventions. These culturally tailored trials included ethnic recipes and language translations in their methods and took place it was predominantly Hispanic/Latino, African American, and Asian communities. These culturally appropriate interventions led to improved outcomes in weight, diet quality, and diabetes control [70].

A concrete example of a program that includes culturally tailored interventions to their outreach is the Centers for Disease Control and Prevention’s Racial and Ethnic Approaches to Community Health (REACH). This initiative is an example of how funding culturally tailored, community-led interventions can reduce chronic disease inequities across racial and ethnic populations. From 2009 to 2012, REACH accomplished significant public health improvements: smoking prevalence declined by 7.5% among non-Hispanic Black adults and by 4.5% among Hispanic adults. Among REACH communities addressing heart disease or diabetes, daily fruit and vegetable intake increased by 3.9% in non-Hispanic Black individuals and by 9.3% in Hispanic individuals. Cholesterol screening rates also rose notably—to 74–78% among African American, 58–71% among Hispanic, and 53–72% among Asian populations—data that demonstrate tangible improvements in health behaviors among minorities [71].

## 9. Discussion

Dietary interventions remain the first-line treatment for MASLD/MASH. Across trials and systematic reviews, Mediterranean and plant-forward patterns consistently reduce intrahepatic fat, while Western dietary patterns—high in red/processed meats, refined carbohydrates, and saturated fats—promote lipotoxicity, hepatic inflammation, and progression risk. Select nutrient-focused strategies (e.g., vitamin E for carefully chosen non-diabetic adults with biopsy-proven disease) can reduce steatosis and lobular inflammation, though antifibrotic effects are less consistent. Food-specific approaches such as resistant starch lower intrahepatic triglycerides, and beans/legumes can beneficially modulate the microbiome and metabolic/inflammatory profiles.

Beyond what is eaten, when it is eaten also matters. Time-restricted eating (TRE) is feasible and can improve weight, metabolic biomarkers, and circadian alignment, complementing calorie restriction and potentially aiding hepatic steatosis. Weight reduction remains the strongest, scalable lever: even modest loss improves steatosis and ALT, and well-structured lifestyle programs can achieve short-term benefits comparable to pharmacologic weight loss.

Mechanistically, the microbiome—via the gut–liver and oral–gut–liver axes—links diet, barrier function, microbial metabolites, and hepatic inflammation/fibrogenesis. While prebiotics/probiotics show mixed effects, synbiotics and exploratory FMT/microbial consortia suggest potential to improve enzymes, stiffness, and lipid profiles in selected settings; sustained benefits likely require coupling microbiome-targeted strategies with weight loss and dietary quality.

Looking ahead, multi-omics and precision nutrition—integrating metagenomics, metabolomics, and epigenomics—are beginning to identify mechanistic signatures (e.g., short-chain fatty acids, bile-acid derivatives) that can stratify patients, monitor response, and guide targeted diet–microbiome interventions. A pragmatic care model thus emphasizes: (1) Mediterranean/plant-forward eating with minimal ultra-processed foods; (2) TRE to reinforce circadian and metabolic health; (3) structured weight-loss support; and (4) microbiome-informed strategies guided by omics-derived biomarkers—while longer, well-controlled trials are needed to confirm histologic and antifibrotic benefits.

Collectively, these findings support an integrative lifestyle medicine framework for MASLD/MASH that positions dietary quality, meal timing, and metabolic–behavioral alignment as interconnected therapeutic pillars. At its core, this model prioritizes Mediterranean and plant-forward dietary patterns as the foundational approach to reduce intrahepatic fat, systemic inflammation, and cardiometabolic risk. Time-restricted eating (TRE) complements this foundation by reinforcing circadian and metabolic homeostasis, while structured weight-loss interventions remain the most effective and scalable means to achieve histologic and biochemical improvement. Microbiome-targeted strategies, when combined with dietary optimization, may further modulate gut–liver signaling and inflammatory tone, offering a mechanistic bridge between nutrition and hepatic resilience. Incorporating multi-omics insights—from metabolomics and metagenomics to epigenomic profiling—can enable a more precision-oriented approach to identify responders and tailor interventions. Importantly, implementation of this framework must integrate equity, accessibility, and cultural adaptability, ensuring that lifestyle medicine for MASLD is both evidence-based and globally relevant. Together, these components define a pragmatic, multidimensional strategy that aligns metabolic, microbial, and behavioral domains to prevent disease progression and promote long-term hepatic and cardiometabolic health.

Scope and limitations. This review is not comprehensive in scope, as it focuses specifically on dietary interventions in MASLD and MASH, rather than covering all chronic liver diseases. It was not conducted as a systematic review and was limited to studies published in English and Spanish, which may have led to the exclusion of relevant research in other languages. Our objective was not to capture every available study but rather to provide a focused overview of the current evidence and highlight future directions for dietary and microbiome-targeted interventions. Additionally, while we prioritized the inclusion of randomized controlled trials, there remains a clear need for more intervention studies specifically focused on dietary interventions.

Equity and implementation. Real-world translation must address access, equity, and cultural relevance. Culturally tailored, community-engaged interventions are critical for adherence and outcomes—particularly in underserved populations disproportionately affected by MASLD/MASH.

## 10. Future Directions and Research Priorities

The study reaches the following conclusions:

- Causal inference at scale: Use target trial emulation and real-world data analytics (active comparators, new-user designs, g-methods) to strengthen causal estimates of dietary interventions and minimize immortal-time and confounding biases.
- Longer, outcome-rich trials: Prioritize ≥48–96-week pragmatic studies with standardized imaging/biomarker panels (e.g., MRI-PDFF, MRE, serologic fibrosis markers) and, when feasible, histology to assess durability and antifibrotic effects.
- Mechanistic depth via multi-omics: Integrate metagenomics, metabolomics, proteomics, and epigenomics with clinical phenotypes to uncover pathways (e.g., SCFAs, bile-acid derivatives) that mediate response and reveal therapeutic targets.
- Microbiome axes: Test the gut–liver and oral–gut–liver pathways by pairing oral health interventions, barrier integrity measures, and microbial/metabolite profiling to evaluate causality and translational potential.
- Personalization: Apply systems biology and machine-learning models to stratify patients by dietary and microbiome response phenotypes, enabling precision nutrition.
- Implementation and sustainability: Evaluate scalable, culturally tailored delivery models (digital tools, community partnerships) to improve adherence and real-world effectiveness.
- Equity: Intentionally include and co-design with underserved populations to address access gaps and ensure generalizability.

## 11. Conclusions

As evidence favoring dietary interventions in MASLD and MASH continues to grow, a shift toward more integrative, mechanism-informed, and personalized approaches is underway. While existing strategies, such as Mediterranean-style diets, time-restricted eating, and microbiome modulation, demonstrate meaningful short-term benefits, sustained impact on fibrosis and long-term outcomes remains a critical research frontier. To move from promise to practice, future efforts must balance scientific rigor with real-world relevance, embedding equity, cultural tailoring, and scalable delivery into the design of next-generation interventions.

## Figures and Tables

**Table 1:** Summary of clinical and mechanistic trials assessing dietary, weigh reduction, and microbiome interventions in MASLD/MASH.

Section	Type of Study	Study (Year, Country)	Population/Inclusion	Final n	Intervention (Type and Dose)	Comparator	Duration	Primary Liver Outcomes and Methods	Main Results	Notes/Risk of Bias
**3.1.1 Mediterranean/plant-forward**	RCT (3-arm)	DIRECT-PLUS (Meir et al., 2021, Israel) [21]	Adults with abdominal obesity/dyslipidemia	294	Green-MED (MED + Mankai + green tea + walnuts; reduced red meat)	Standard MED; healthy dietary guidelines	18 mo	Intrahepatic fat by MRI-PDFF (primary)	Green-MED: IHF −38.9%; MED −19.6%; Guidelines −12.2%; NAFLD prevalence ≈ 50% lower.	Randomized; imaging blinded; behavioral counseling in all arms
**3.1.3 Ketogenic/Low-Carbohydrate**	RCT (3-arm, open-label)	Holmer et al., 2021 (Sweden) [25]	Adults with MASLD (MRI/MRS-confirmed steatosis)	74	Low-carb high-fat (LCHF) diet, ad libitum, dietitian-guided	5:2 intermittent calorie restriction; standard hepatology lifestyle advice (SoC)	12 wks	Intrahepatic fat by ^1^H-MRS (primary); TE stiffness; metabolic markers	Both LCHF and 5:2 reduced IHTG vs. SoC (Δ LCHF −7.2% [95% CI −9.3, −5.1]; 5:2 −6.1% [−8.1, −4.2]; SoC −3.6% [−5.8, −1.5]); mean weight loss ≈ 7 kg; liver stiffness improved in 5:2 and SoC but not in LCHF.	Open-label; behavioral counseling in all arms; short duration; MRI blinded
	Pilot RCT (open-label)	Chirapongsathorn et al., 2025 (Thailand) [28]	Adults with MASLD	24	Home-delivery ketogenic diet program (very-low-carb, KD)	DASH-based nutrition education/standard lifestyle advice	8 wks	Steatosis and stiffness by transient elastography (CAP/LSM); body weight; metabolic risk factors	No significant change in steatosis or stiffness; KD led to greater weight loss (−6.16 kg vs. −2.14 kg) and reductions in waist circumference, fat mass, triglycerides, and systolic blood pressure; HDL decreased in the KD arm	Small sample; short duration; elastography (not MRI-PDFF); home-delivery support likely increased adherence
	Mechanistic trial (pre–post)	Luukkonen et al., 2020, (Finland/USA) [27]	Adults with NAFLD, overweight/obesity	10	Strict ketogenic diet (~6 days; <20 g carb/day) to induce nutritional ketosis	Baseline (self-controlled)	6 days	IHTG by ^1^H-MRS (primary); hepatic insulin resistance; isotope-traced mitochondrial flux (NMR/LC-MS)	IHTG decreased by 31% with 3% weight loss; hepatic insulin resistance decreased by 58%, with greater β-oxidation and lower de novo lipogenesis. IFC change −7.7% (T1) vs. −2.6% (T3, *p* = 0.047); Mediterranean-diet adherence increased by 5.2 points; fibrosis and stiffness were unchanged	Very short term; small N; mechanistic focus; no control arm; robust fluxomics data
**3.1.4 Ultra-processed foods (UPF)**	Longitudinal cohort (secondary analysis within FLIPAN)	García et al., 2025, Spain [36]	Adults 40–60 y; BMI 27–40 kg/m^2^; MRI-diagnosed MASLD; ≥3 MetS criteria (IDF)	70	Δ UPF (NOVA) across tertiles (T1 max reduction ≤ −7.27 pp vs. T3 min ≥ −0.62 pp)	Between-tertile comparisons	6 mo	Intrahepatic fat by MRI; US steatosis; FibroScan stiffness	IFC −7.7% (T1) vs. −2.6% (T3), *p* = 0.047; Mediterranean-diet adherence increased 5.2 points (T1); fibrosis/stiffness NS	Secondary analysis; small n
**3.1.5 Nutrient-specific—vitamin E**	Multicenter RCT, double-blind	PIVENS (Sanyal et al., 2010, USA) [39]	Non-diabetic, biopsy-proven NASH	247	α-tocopherol 800 IU/day	Placebo (and pioglitazone arm)	96 wks	Histology (NAS components; fibrosis stage)	Vitamin E improved steatosis, ballooning, and inflammation vs. placebo; no clear antifibrotic effect	Strong histology endpoint; non-diabetic only
**3.1.5 Nutrient-specific—n-3 PUFA**	RCT (double-blind)	Cansanção et al., 2020, Brazil [40]	Adults with US-NAFLD	24	Fish oil ≈ 1509 mg DHA + 306 mg EPA/day	Olive-oil placebo	6 mo	Liver stiffness (FibroScan); CAP; biomarkers	Decreased Stiffness (*p* = 0.039) and ALP (*p* = 0.002); RBC DHA increased (adherence)	Pilot size; elastography (not biopsy)
**3.1.6 Food-specific—resistant starch**	RCT (double-blind)	Ni et al., 2023, China [43]	Adults with MRI-quantified NAFLD	196	Resistant starch 40 g/day	Isocaloric control starch	4 mo	IHTG by MRI-PDFF (primary); microbiome and metabolites	Absolute IHTG −9.1% vs. control; favorable microbiome and BCAA shifts	Strong mechanistic profiling
**3.1.6 Food-specific—beans (legumes)**	RCT (parallel)	Zhang et al., 2023, USA (“BE GONE”) [44]	Adults with overweight/obesity and colorectal neoplasia risk	55	High-bean diet	Control	8 wks	Microbiome and metabolic biomarkers	Beans increased beneficial metabolites and induced favorable gut-microbiota changes.	Not a NAFLD sample; gut–liver axis relevance
**4. Meal timing (TRE/TRF)**	RCT (parallel)	TREATY-FLD (Wei et al., 2023, China) [47]	Adults with obesity and NAFLD	88	8 h TRE (08:00–16:00) + caloric targets	Daily calorie restriction (habitual timing)	12 mo	IHTG by MRI (primary); body fat and metabolic risk	IHTG decreased by 6.9% (TRE) and 7.9% (DCR); no added benefit of TRE under isocaloric conditions.	Strong design; energy restriction likely main driver
	RCT (single-blind)	Feehan et al., 2023, Australia [46]	Adults with NAFLD	32	16:8 TRF (no calorie targets)	Standard care	12 wks	CAP steatosis; visceral adiposity	Time-restricted feeding reduced CAP and visceral fat versus standard advice (short-term).	Pilot scale; CAP endpoint
**5. Body-weight reduction**	RCT	Khoo et al., 2019 (Liver Int), Singapore [48]	Obese, non-diabetic NAFLD; MRI-LFF ≥ 5.5%	30	Liraglutide 3.0 mg/day	Structured lifestyle (~500 kcal/d deficit)	12–26 wks + f/u	Liver fat by MRI; ALT/AST; weight	Similar weight loss (≈−3.5 kg) and LFF reductions (~−7% to −9%); ALT/AST improved in both arms	Highlights weight loss as primary driver
	Randomized experimental-medicine trial	Moolla et al., 2025, UK [49]	MASLD; no T2D; phenotyping baseline–12 wks–12 wks off	29	GLP-1RA (liraglutide; dose not specified)	Lifestyle (~500 kcal/d deficit; matched weight loss)	12 wks + 12 wks off	Liver fat by MRS; cT1; ALT/AST; multi-omics	Matched weight loss both arms; similar reductions in liver fat and ALT; GLP-1RA improved glucose/lipid benefits; post-withdrawal proteomic rebound	Small, open-label; short duration; industry links declared
**6.1 Prebiotics**	Pilot RCT	Reshet et al., 2024, Israel [50]	Adults 18–70 y, MASLD + MetS, ALT ≥ 30 U/L	19	Prebiotic supplement (weight stable)	Placebo	8–12 wks	Liver fat; hepatic/metabolic markers; FGF-19; fecal taxa	Increased *Bifidobacterium* but no liver/metabolic benefit without weight loss	Underpowered for clinical endpoints
**6.1 Probiotics**	RDBPC RCT	Ahn et al., 2019, Korea [51]	Adults with NAFLD, MRI-PDFF > 5%	68	6-strain mix (*L. acidophilus*, *L. rhamnosus*, *L. paracasei*, *Pediococcus pentosaceus*, *B. lactis*, *B. breve*)	Placebo	12 wks	MRI-PDFF (IHF %); labs	IHF reduction 2.6% vs. placebo (*p* = 0.012); TG −34 mg/dL; weight loss confounded some effects	Good imaging endpoint; short duration
**6.3 FMT vs. probiotics**	RCT	Xue et al., 2022, China [53]	Adults with NAFLD	75	FMT (colonoscopy) + 3 enemas over 3 days + lifestyle advice	Oral probiotics + same advice	1 mo	Hepatic fat; metabolic indices	FMT reduced hepatic fat vs. probiotic control at 1 mo	Very short follow-up; co-interventions present
**6.4 Oral–gut–liver (periodontal therapy)**	Multicenter RCT	PERION (Kamata et al., 2022, Japan) [57]	NAFLD + periodontitis; ALT ≥ 40 U/L; steatosis grade ≥ 1	40	Scaling and root planing (SRP)	Usual care/tooth-brushing	12 wks	ALT (primary); *P. gingivalis* IgG; endotoxemia	SRP reduced ALT and *P. gingivalis* titers vs. control	Pragmatic dental intervention; biochemical outcomes

Abbreviations: MRI-PDFF, magnetic resonance imaging–proton density fat fraction; IHF, intrahepatic fat fraction; NAFLD, nonalcoholic fatty liver disease; MASLD, metabolic dysfunction–associated steatotic liver disease; MRS, magnetic resonance spectroscopy; SoC, standard of care; LCHF, low-carbohydrate high-fat diet; KD, ketogenic diet; CAP, controlled attenuation parameter; LSM, liver stiffness measurement; HDL, high-density lipoprotein cholesterol; IHTG, intrahepatic triglyceride content; IFC, intrahepatic fat change; T1/T3, tertile 1/tertile 3; NMR, nuclear magnetic resonance; LC-MS, liquid chromatography–mass spectrometry; UPF, ultra-processed foods; FLIPAN, Fatty Liver in the PREDIMED-Plus Ancillary Network; IDF, International Diabetes Federation; NS, not significant; NAS, NAFLD activity score; n-3 PUFA, omega-3 polyunsaturated fatty acids; DHA, docosahexaenoic acid; EPA, eicosapentaenoic acid; BCAA, branched-chain amino acids; TRE, time-restricted eating; DCR, daily calorie restriction; TRF, time-restricted feeding; LFF, liver fat fraction; GLP-1RA, glucagon-like peptide-1 receptor agonist; cT1, corrected T1 mapping (MRI-based fibrosis surrogate); FMT, fecal microbiota transplantation; SRP, scaling and root planing; *P. gingivalis*, *Porphyromonas gingivalis*.

## Data Availability

No new data were created or analyzed in this study. Data sharing is not applicable to this article.
